# COVID-19 awareness, knowledge and perception towards digital health in an urban multi-ethnic Asian population

**DOI:** 10.1038/s41598-021-90098-6

**Published:** 2021-05-24

**Authors:** Cong Ling Teo, Miao Li Chee, Kai Hui Koh, Rachel Marjorie Wei Wen Tseng, Shivani Majithia, Sahil Thakur, Dinesh Visva Gunasekeran, Simon Nusinovici, Charumathi Sabanayagam, Tien Yin Wong, Yih-Chung Tham, Ching-Yu Cheng

**Affiliations:** 1 Singapore Eye Research Institute, Singapore National Eye Centre, The Academia, 20 College Road, Discovery Tower Level 6, Singapore, 169856 Singapore; 2 Duke-NUS Medical School, Singapore, Singapore; 3 Department of Ophthalmology, Yong Loo Lin School of Medicine, National University of Singapore and National University Health System, Singapore, Singapore

**Keywords:** Epidemiology, Patient education

## Abstract

This study aimed to determine COVID-19-related awareness, knowledge, impact and preparedness among elderly Asians; and to evaluate their acceptance towards digital health services amidst the pandemic. 523 participants (177 Malays, 171 Indians, 175 Chinese) were recruited and underwent standardised phone interview during Singapore’s lockdown period (07 April till 01 June 2020). Multivariable logistic regression models were performed to evaluate the associations between demographic, socio-economic, lifestyle, and systemic factors, with COVID-19 awareness, knowledge, preparedness, well-being and digital health service acceptance. The average perception score on the seriousness of COVID-19 was 7.6 ± 2.4 (out of 10). 75.5% of participants were aware that COVID-19 carriers can be asymptomatic. Nearly all (≥ 90%) were aware of major prevention methods for COVID-19 (i.e. wearing of mask, social distancing). 66.2% felt prepared for the pandemic, and 86.8% felt confident with government’s handling and measures. 78.4% felt their daily routine was impacted. 98.1% reported no prior experience in using digital health services, but 52.2% felt these services would be helpful to reduce non-essential contact. 77.8% were uncomfortable with artificial intelligence software interpreting their medical results. In multivariable analyses, Chinese participants felt less prepared, and more likely felt impacted by COVID-19. Older and lower income participants were less likely to use digital health services. In conclusion, we observed a high level of awareness and knowledge on COVID-19. However, acceptance towards digital health service was low. These findings are valuable for examining the effectiveness of COVID-19 communication in Singapore, and the remaining gaps in digital health adoption among elderly.

## Introduction

As of end January 2021, the coronavirus disease 2019 (COVID-19) has infected approximately 100 million people, leading to more than 2 million deaths globally^1^. This global pandemic has brought upon unprecedented challenges worldwide. Given the rapid spread of COVID-19, a society’s awareness and knowledge of COVID-19, key preventative measures (e.g., wearing masks) and acceptance of new models of care (e.g., video-consultations)^2^ is important. Many communication channels have been concurrently swamped by a deluge of misinformation (hence, dubbed an “infodemic”)^3^, further highlighting the need for policy makers and healthcare providers to provide reliable information that is easily understood and accessible^4,5^. Adequate health literacy amongst the general population is also critical to complement government-implemented measures. Having a high level of awareness about recommended preventive health behaviour (e.g., mask wearing, hand hygiene) are essential safeguards against the community spread of COVID-19. All of these can significantly improve disease prevention, timely diagnosis and management.

Recent studies indicated that older adults and those with systemic comorbidities (e.g. cardiovascular diseases, cancer, diabetes, hypertension) are more vulnerable to serious complications and death caused by COVID-19 infection^6–8^. Given that elderly are also more prone to having these health comorbidities, the mortality risk of COVID-19 would be higher in elderly population than middle-aged or younger individuals^8–10^. Hence, there is a greater urgency to ensure elderly population have a high level of awareness on prevention against COVID-19.

Despite the higher risk of COVID-19 in the elderly population and typically lower healthy literacy levels in general among the elderly^11–14^, few studies have evaluated the health literacy levels of the elderly on COVID-19 especially in Asian population^4,15,16^. In brief, the studies conducted in Western countries found that a substantial number of participants had low health literacy levels with some citing difficulties in judging the myriad of information available online and low levels of preparedness for the pandemic outbreak^4,15^. On the other hand, the study conducted in China found high levels of preventive behaviours among study participants. However, this study mainly constituted of young participants (mean age = 31 years, only 25 were aged ≥ 60 years)^16^. Taken together, the impact of COVID-19 on the well-being of elderly population has not been investigated comprehensively in Asians.

On the other hand, digital health services have gradually played bigger roles in minimizing face-to-face contact between physicians and patients and thus reducing non-essential commuting in the community^17^. However, to date the perception and acceptance of digital health services have not been well-documented among Asian elderly population as well. As we move into a new normal post COVID-19, the shift of trend towards digital health services (e.g. teleconsultation with doctor; medical chatbot, or using artificial intelligence (AI) to review medical report) will gradually become more common^17^.

The aim of this study was two-fold. Firstly, to determine COVID-19-related awareness, knowledge, impact and preparedness among elderly Asian adults. Secondly, to evaluate their acceptance towards digital health services amidst the pandemic. Findings from this study would provide useful insights to aid development and design of COVID-19 related public measures, as we enter a new normal post COVID-19.

## Methods

### Study population

Participants were recruited from the Singapore Epidemiology of Eye Diseases (SEED) cohort study. The SEED cohort is a population-cohort study comprising of the three major ethnic groups in Singapore (Malays, Indians and Chinese)^18–22^. Participants aged 60 years and above were selected for the questionnaire. Verbal informed consent was obtained from all study participants by a trained study team over the phone because the questionnaire was performed during Singapore’s nationwide lockdown period (07 April to 1 June 2020), whereby only essential business and service providers were allowed to open e.g., food stalls, supermarkets, hospitals, utilities and transport; schools were moved to full home-based learning, restrictions were imposed on movements and social gatherings. All study procedures were conducted in accordance with the Declaration of Helsinki and approved by the SingHealth Centralized Institutional Review Board (CIRB reference number 2020/2356).

### SEED COVID-19 Questionnaire

SEED COVID-19 Questionnaire was adapted from the Chicago COVID-19 Comorbidities (C3) Survey conducted in the United States during March 2020^4^. The SEED COVID-19 questionnaire investigated elements related to COVID-19, including participants’ awareness, concern, knowledge, and preparedness. Additionally, the SEED COVID-19 questionnaire assessed the impacts of COVID-19 on general health well-being, and participants’ acceptance of digital health services.

### COVID-19 awareness and concern

Participants were asked in their preferred languages (English, Malay, Tamil or Mandarin) to rate the gravity of COVID-19 as a public health threat (on a scale of 1 to 10, 1 being “No threat at all” and 10 being “Very serious threat”). In addition, participants were assessed on their worries about contracting COVID-19 (“Very worried”, “A little worried”, or “Not worried at all”) and if they thought they would get sick from COVID-19 (“I definitely will”, “I probably will”, “Neutral” or “Not at all”). The last question in this section evaluated participants’ opinions on the likelihood that someone they knew would get sick from the disease this year ("Very likely”, “Somewhat likely”, “Neutral”, “Somewhat unlikely”, or “Not at all likely”).

### COVID-19 knowledge

The SEED COVID-19 questionnaire also investigated participants’ knowledge on the presence of asymptomatic COVID-19 carriers (“Yes”, “Unsure” or “No”). Participants were asked to rate the likelihood of a COVID-19 infected person displaying mild/no clinical symptoms and whether an infected person would die as a result, on a scale of 1 to 10 (1 being “Not at all likely”, 10 being “Very likely”). Five COVID-19 prevention methods (1: Wear a mask as long as leaving house; 2: Keep a minimum one metre apart from public members; 3: Wash hands frequently; 4: Stay at home as much as possible; 5: Avoid touching face, eye, nose and mouth) were listed individually and participants were asked for their views on the importance of these five methods. (“Yes”, “Unsure” or “No”).

### Information sources

Participants’ sources of information on COVID-19 were determined from a list of potential sources (“Television”, “Newspaper”, “Radio”; “Family and Friends”; “Posters/Leaflets; “Hospital/Polyclinic/General Practitioner”; “Internet/Sharing on social media”, and “Sharing on messaging applications”).

### Confidence in government and individual preparedness level

Participants’ confidence in the Singapore government’s ability to prevent further outbreak in the local community (“Very confident”, “Somewhat confident”, “Neutral”, “Not very confident”, or “Not confident at all”) along with their preparedness in the event of another outbreak in the community (“Very prepared”, “Somewhat prepared”, “Neutral”, “Not very well prepared”, or “Not prepared at all”) were evaluated.

### General health and well-being

Participants were asked to rate how much their daily routines had been impacted due to COVID-19 (“A lot”, “Moderate”, “A little”, or “Not at all”). “A change in daily routine” was recorded as ‘yes’ when participants responded with either “a lot”, “moderate” or “a little”.

The impact on participants’ general health was assessed by asking participants if they experienced any of the following symptoms: “Lose much sleep”, “Feel under stress”, “Feel unable to ‘face up’ to problems encountered”, and “Feel unhappy/depressed” over the past one month in comparison to their normal routine. The response options were “Less than usual”, “No more than usual”, “Rather more than usual”, or “Much more than usual”. Well-being affected was defined as ‘yes’ when participants had at least one response with “Rather more than usual” or “Much more than usual” to the symptoms.

### Individual acceptance towards digital health

Participants’ acceptance towards digital health services (“Video consultation with doctors”; “Messaging applications with doctors”; “Artificial Intelligence (AI) software or Computer for screening of any health condition”; “Online Questions & Answers platforms”) was evaluated by asking if participants had used any of these services before COVID-19 outbreak. Participants were also asked on the helpfulness of these digital health services in reducing non-essential contact between patients and health care providers (“Yes”, “Unsure” or “No”) and the likelihood of them using these services (“Very likely”, “Somewhat likely”, “Neutral”, “Somewhat unlikely”, or “Not at all likely”). Participants’ comfort level in using software/AI system to review medical reports and provide advice automatically was evaluated too (“Very comfortable”, “Somewhat comfortable”, “Neutral”, “Somewhat uncomfortable” or “Not at all comfortable”).

### Other measurements and systemic assessments

Body-mass index (BMI) was calculated as body weight (in kilograms) divided by body height (in meters) squared. Blood pressure (BP) was measured using a digital automated BP monitor (Dinamap model Pro Series DP110X-RW, 100V2; GE Medical Systems Information Technologies Inc., Milwaukee, USA). Hypertension was defined as systolic blood pressure (BP) ≥ 140 mmHg, diastolic BP ≥ 90 mmHg, physician’s diagnosis, use of hypertensive medication and/or self-reported history of hypertension. Diabetes mellitus (DM) was defined as random glucose ≥ 11.1, glycosylated haemoglobin (HbA1C) ≥ 6.5%, use of diabetic medication(s) and/or self-reported history. Kidney function was assessed using estimated glomerular filtration rate (eGFR) from serum creatinine using the chronic kidney disease epidemiology collaboration (CKD-EPI) equation^18^. Subjects were defined to have chronic kidney disease (CKD) if GFR was less than 60 mL/min/1.73m^2^.

Non-fasting venous blood samples were collected for biochemistry tests including plasma cholesterol [total cholesterol, low-density lipoprotein (LDL) and high-density lipoprotein (HDL)], serum triglyceride (TG), HbA1c, creatinine, and random glucose.

Interviewer-administered questionnaires conducted in the participant’s language of choice were utilized in order to obtain participant information on their demographic, lifestyle factors and medical history such as smoking status, weekly alcohol intake, monthly income (earning less than or more than/equals to S$2000), education level (no formal education or formal education i.e. primary school educated and above) and housing type (1–2 room flat, 3–4 room flat or 5-room public housing flat/private housing). Cardiovascular disease (CVD) was defined as self-reported myocardial infarction, angina or stroke.

### Statistical analysis

All statistical analyses were performed using Stata 14.0 (StataCorp LP, College Station, TX). For descriptive statistics, the mean and standard deviation were reported for continuous demographic characteristics and COVID-19 survey responses by ethnic groups, while frequency and percentage were reported for categorical characteristics. Multivariable logistic regression models were performed to evaluate the associations between demographic factors (age, gender, ethnicity), socio-economic factors (income level, education level, housing type, living alone), lifestyle (smoking), and history of chronic systemic diseases (diabetes, hypertension, chronic kidney disease, CVD or hyperlipidaemia), with COVID-19 awareness, knowledge, preparedness, well-being and acceptance towards digital health services, respectively.

## Results

We had contacted and invited 745 participants to take part in the questionnaire. A total of 222 participants had rejected to take part, and 523 participants (177 Malay, 171 Indian, and 175 Chinese elderly; response rate of 70.2%) completed the questionnaire. Among the participants, 51.4% were female, 92.4% had formal education and majority of them (81%) had history of hypertension. The mean age was 72.3 ± 7.7 years (Table 1).

**Table 1:** Characteristics of included participants.

Variables	Overall (n = 523)
Age, year	72.3 (7.7)
Female Gender	269 (51.4)
**Ethnicity**	
Malay	177 (33.8)
Indians	171 (32.7)
Chinese	175 (33.5)
Income category (> SGD^+^$2000)	100 (23.6)
Formal education^#^	483 (92.4)
**Housing Category**	
1–2 room public housing flat	33 (6.3)
3–4 room public housing flat	320 (61.3)
≥ 5-room public housing flat	169 (32.4)
Current smoking status, yes	50 (9.6)
Living alone, yes	31 (5.9)
**Presence of chronic systemic diseases**	
Diabetes	189 (36.1)
Hypertension	421 (81.0)
Chronic kidney disease	83 (17.8)
Cardiovascular disease*	75 (14.3)
Hyperlipidaemia	334 (66.9)

Data presented are mean (standard deviation) or frequency (percentage), where appropriate.

^+^SGD = Singapore Dollar.

^#^Defined as having primary or higher education.

*Defined based on self-reported history of stroke, heart attack or angina.

### COVID-19 awareness and concern

Table 2 presents participants’ responses to questionnaires pertaining to COVID-19 awareness, knowledge, preparedness, well-being and acceptance towards digital health services. Among the participants, the mean score on the perceived seriousness of COVID-19 was 7.6 ± 2.4 (out of 10, Table 2a). 33.5% (n = 175) participants responded being “very worried” about getting COVID-19, 35.2% (n = 184) stating that they were “a little worried” and the remaining 31.4% (n = 164) participants expressed that they were “not worried at all” (Table 2a). Across ethnicity, among the 191 participants who perceived that they “will not get sick from COVID-19" (Table 2a), 58.1% (n = 111) were Indians, 20.9% (n = 40) were Malays and 20.9% (n = 40) were Chinese (Supplementary Table 1a). About 56.7% (n = 97) Indian participants responded “not worried at all”, compared to 18.1% (n = 32) of Malay and 20.0% (n = 35) of Chinese participants. Similarly, when asked about how likely someone whom participants knew might get sick from COVID-19, 213 participants responded “not at all likely”, of which 62.9% (n = 134) were Indians, 16.9% (n = 36) were Malays and 20.2% (n = 43) were Chinese (Supplementary Table 1a). In multivariable analysis, Indian elderly were more likely to “not feel worried about getting COVID-19” as compared to Malays (odds ratio [OR] = 5.37; P < 0.001; Table 3).

**Table 2:** Knowledge, attitude, preparedness and digital health acceptance toward COVID-19.

	Overall (n = 523)
**(a) COVID-19 awareness and concern**	n (%)/mean ± SD
On a scale of 1 to 10, how serious of a public health threat do you think the COVID-19 is or might become? (1 being no threat at all, 10 being a very serious public health threat)	7.6 ± 2.4
**How worried are you about getting the COVID-19?**	
Very worried	175 (33.5)
A little worried	184 (35.2)
Not worried at all	164 (31.4)
**Do you think that you will get sick from the COVID-19 ?**	
I definitely will	81 (15.5)
I probably will	113 (21.6)
Neutral	138 (26.4)
Not at all	191 (36.5)
**How likely it is that someone you know may get sick from the COVID-19 this year?**	
Very likely	34 (6.5)
Somewhat likely	60 (11.5)
Neutral	156 (29.8)
Somewhat unlikely	60 (11.5)
Not at all likely	213 (40.7)
**(b) COVID-19 knowledge**	
**Are you aware that COVID-19 carriers can be asymptomatic? For example, absence of running nose, cough, fever or appearing to be fine**	
Yes	395 (75.5)
Unsure	77 (14.7)
No	51 (9.8)
On a scale of 1 to 10, how likely do you think a person who is infected by COVID-19 will display no symptoms or mild symptoms? e.g. mild cough, itchy throat and mild fever. (1 being not at all likely, 10 being a very likely)	5.3 ± 2.3
On a scale of 1 to 10, how likely do you think a person who get COVID-19 will die as a result? (1 being not at all likely, 10 being a very likely)	5.1 ± 2.7
**Which of the following do you think are important prevention methods for the COVID-19?:**	
**Wear a mask (as long as you are outside of the house)**	
Yes	511 (97.7)
Unsure	3 (0.6)
No	9 (1.7)
**Keep a minimum distance of 1 m from others in the public**	
Yes	502 (96.0)
Unsure	5 (1.0)
No	16 (3.1)
**Wash your hands frequently**	
Yes	509 (97.3)
Unsure	3 (0.6)
No	11 (2.1)
**Stay at home as much as possible**	
Yes	492 (94.1)
Unsure	9 (1.7)
No	22 (4.2)
**Avoid touching your face, eyes, nose and mouth**	
Yes	483 (92.4)
Unsure	13 (2.5)
No	27 (5.2)
**(c) Information sources on COVID-19**	
**Where do you get information about COVID-19? (Check all that apply)**	
TV	472 (90.2)
Newspaper	260 (49.7)
Radio	177 (33.8)
Family and Friends	208 (39.8)
Posters/Leaflets	29 (5.5)
Hospital/Polyclinic/GP	14 (2.7)
Internet/ Sharing on Social Media (Facebook/Twitter/etc.)	136 (26.0)
Sharing on messaging applications (Whatsapp/ etc.)	120 (22.9)
**(d) Confidence in Government and Individual Preparedness**	
**How confident are you that Singapore government can prevent a further widespread outbreak in the local community?**	
Very confident	363 (69.4)
Somewhat confident	91 (17.4)
Neutral	56 (10.7)
Not very confident	8 (1.5)
Not confident at all	5 (1.0)
**How prepared do you think you are if there were to be a further widespread outbreak in the local community?**	
Very prepared	193 (36.9)
Somewhat prepared	153 (29.3)
Neutral	138 (26.4)
Not very well prepared	22 (4.2)
Not prepared at all	17 (3.3)
**(e) Impact of COVID-19 pandemic on General Health and Well-being**	
**How much has the COVID-19 pandemic changed your daily routine?**	
A lot	159 (30.4)
Moderate	112 (21.4)
A little	139 (26.6)
Not at all	113 (21.6)
**How much has the COVID-19 pandemic caused you to lose sleep?**	
Less than usual	57 (10.9)
No more than usual	410 (78.4)
Rather more than usual	44 (8.4)
Much more than usual	12 (2.3)
**How much has the COVID-19 pandemic caused you to feel under stress?**	
Less than usual	62 (11.9)
No more than usual	363 (69.4)
Rather more than usual	75 (14.3)
Much more than usual	23 (4.4)
**How much has the COVID-19 pandemic caused you to feel ‘unable to face up’ to problems encountered?**	
Less than usual	59 (11.3)
No more than usual	421 (80.5)
Rather more than usual	30 (5.7)
Much more than usual	13 (2.5)
**Feel unhappy and depressed**	
Less than usual	64 (12.2)
No more than usual	363 (69.4)
Rather more than usual	78 (14.9)
Much more than usual	18 (3.4)
**(f) Assessment on Individual Acceptance towards Digital Health, Pre and Post COVID-19**	
**Before the COVID-19 outbreak, have you used any of these ‘digital medical services’ for medical consultation or follow-up? (Check all that apply)**	
None at all	513 (98.1)
Messaging applications with doctors (for example, Whatsapp/ SMS text chat)	8 (1.5)
Video consultation with doctors	3 (0.6)
**Do you agree that the digital medical services mentioned in the previous question may be helpful to reduce non-essential contact between patients and doctors/health care providers?**	
Yes	273 (52.2)
Unsure	143 (27.3)
No	107 (20.5)
**If the COVID-19 pandemic continues, how likely will you use these digital medical services (video consultation with doctors, Whatsapp/ SMS text chat)**	
Very likely	49 (9.4)
Somewhat likely	83 (15.9)
Neutral	100 (19.1)
Somewhat unlikely	33 (6.3)
Not at all likely	258 (49.3)
**If the COVID-19 pandemic continues, will you feel comfortable using automated software/AI systems to interpret your medical tests/scans and provide advice automatically?**	
Very comfortable	48 (9.2)
Somewhat comfortable	68 (13.0)
Neutral	102 (19.5)
Somewhat uncomfortable	41 (7.8)
Not at all comfortable	264 (50.5)

**Table 3:** Association between demographic, socio-economic factors and medical history with levels of concern, preparedness and behaviours related to COVID-19.

Factors	Concern		Preparedness		Related behaviours			
	Not Worried		Not Prepared		Change of daily routine^+^		Well-being affected*	
	OR (95%CI)	P	OR (95%CI)	P	OR (95%CI)	P	OR (95%CI)	P
Age, per 5 years older	1.18 (0.99–1.39)	0.061	1.16 (0.89–1.53)	0.273	**0.75 (0.62**–**0.91)**	0.004	0.88 (0.73–1.04)	0.135
Female gender	1.03 (0.63–1.69)	0.892	1.49 (0.66–3.37)	0.340	0.64 (0.36–1.14)	0.132	1.19 (0.72–1.95)	0.498
**Ethnicity**								
Malay	Reference		Reference		Reference		Reference	
Indian	**5.37 (3.03–9.53)**	< 0.001	2.10 (0.68–6.50)	0.200	**0.37 (0.20–0.70)**	0.002	**2.93 (1.45–5.92)**	0.003
Chinese	1.00 (0.54–1.83)	0.992	**3.50 (1.21–10.10)**	0.021	**2.28 (1.09–4.76)**	0.028	**6.78 (3.46–13.29)**	< 0.001
**Income Category**								
< SGD $2000	Reference		Reference		Reference		Reference	
≥ SGD $2000	1.01 (0.55–1.87)	0.972	0.85 (0.28–2.55)	0.770	**3.06 (1.27–7.36)**	0.012	0.90 (0.49–1.64)	0.724
Formal Education^**#**^	1.24 (0.50–3.08)	0.640	0.90 (0.26–3.20)	0.877	1.96 (0.81–4.71)	0.134	0.78 (0.30–1.99)	0.601
**Housing category**								
1–2 room public housing flat	Reference		Reference		Reference		Reference	
3–4 room public housing flat	2.11 (0.64–6.90)	0.219	0.74 (0.15–3.58)	0.706	1.73 (0.61–4.92)	0.307	1.94 (0.51–7.31)	0.330
≥ 5-room public housing flat	1.65 (0.47–5.79)	0.435	0.53 (0.09–2.99)	0.474	1.13 (0.36–3.58)	0.830	2.31 (0.58–9.12)	0.233
Current smoking status, yes	0.81 (0.36–1.84)	0.621	1.70 (0.50–5.78)	0.396	1.09 (0.39–3.00)	0.872	1.47 (0.66–3.27)	0.348
Living alone, yes	1.00 (0.37–2.65)	0.995	0.76 (0.16–3.59)	0.728	0.84 (0.29–2.46)	0.755	0.93 (0.36–2.42)	0.881
History of any chronic systemic diseases^**+**^**, yes**	0.59 (0.26–1.32)	0.200	1.47 (0.32–6.77)	0.623	0.76 (0.24–2.44)	0.642	0.56 (0.26–1.21)	0.141

SGD = Singapore Dollar.

^+^Answered either ‘A Little’, ‘Moderate’ or ‘A lot’ on change in daily routine.

*Answered either ‘More than usual’ or ‘Much more than usual’, for at least one of the questions on well-being impacted.

^#^Defined as having primary or higher education.

^+^Defined as having diabetes, hypertension, chronic kidney disease, cardiovascular disease or hyperlipidaemia.

### COVID-19 knowledge

Of the participants, 75.5% (n = 395) know that COVID-19 carriers can be asymptomatic; and ≥ 92% of them agreed that the listed five prevention methods are important in mitigating transmission of COVID-19 (Table 2b). On average, participants reported a perception score of 5.3 ± 2.3 (out of 10) on the likelihood for “infected person displaying no or mild symptoms” (Table 2b). On the likelihood perception of whether COVID-19 infected person will die from the COVID-19, Malay participants reported a perception score of 6.3 ± 2, compared 5.5 ± 2.5 in Chinese and 3.4 ± 2.8 in Indians (Supplementary Table 1b). Multivariable analysis further showed that, participants residing in smaller public housing flats were more likely to be unaware that COVID-19 carriers can be asymptomatic (OR = 4.25; P = 0.037) (Table 4).

**Table 4:** Associations between demographic, socio-economic factors and medical history with knowledge level on COVID-19 transmission.

Factors	Unaware that COVID-19 carrier can be asymptomatic	
	OR (95%CI)	P
Age (per 5 year increase)	1.01 (0.79–1.30)	0.922
Gender, Female	0.76 (0.35–1.62)	0.477
**Ethnicity**		
Malay	Reference	
Indian	1.87 (0.76–4.59)	0.172
Chinese	1.31 (0.53–3.27)	0.559
**Income Category**		
≥ SGD $2000	Reference	
< SGD $2000	1.43 (0.51–4.00)	0.50
**Education**		
Formal Education^#^	Reference	
No Formal Education	2.60 (0.84–8.08)	0.098
**Housing Category**		
≥ 5-room public housing flat	Reference	
3–4 room public housing flat	1.53 (0.63–3.70)	0.349
1–2 room public housing flat	**4.25 (1.09–16.54)**	0.037
Current smoking status, yes	0.61 (0.16–2.33)	0.471
Living alone, yes	1.80 (0.54–5.94)	0.336
History of any chronic systemic diseases^+^, yes	0.64 (0.20–2.06)	0.456

SGD = Singapore Dollar.

^#^Defined as having primary or higher education.

^+^Defined as having diabetes, hypertension, chronic kidney disease, cardiovascular disease or hyperlipidaemia.

### Information sources on COVID-19

**Figure 1:**
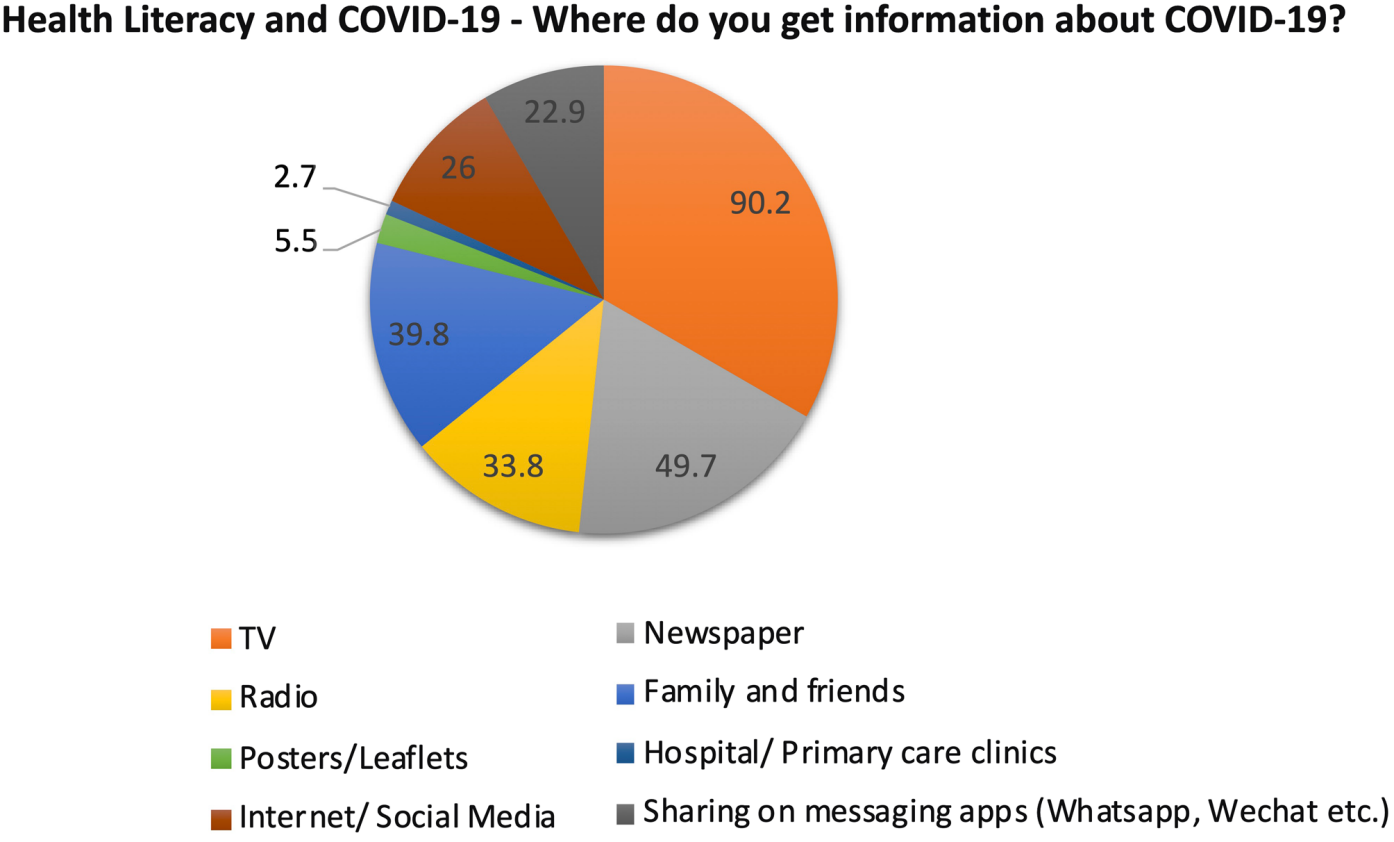
Variety of sources from which respondents get information on COVID-19.

Source of COVID-19 information was obtained mostly from: TV (n = 472, 90.2%), newspaper (n = 260, 49.7%), family and friends (n = 208, 39.8%) and radio (n = 177, 33.8%). Less information was obtained through smart device applications such as internet, social media (n = 136, 26.0%) and messaging application (n = 120, 22.9%). While the least information was obtained through hospital/polyclinic/GP (n = 14, 2.7%) and posters/leaflets (n = 29, 5.5%) (Fig. 1; Table 2c).

### Confidence in government and individual preparedness level

Of the participants, 86.8% (n = 454) were “very confident” or “somewhat confident” with government in preventing a further widespread outbreak in the local community; and 66.2% of the respondents (n = 346) reported “very prepared” or “somewhat prepared” if a widespread outbreak happens locally (Table 2d; Supplementary Figure 3). Multivariable analysis further showed that, compared to Malays, Chinese participants were more likely to expressed feeling unprepared if there were a further widespread (OR = 3.50; P = 0.021) (Table 3).

### General health and well-being questionnaire

**Figure 2:**
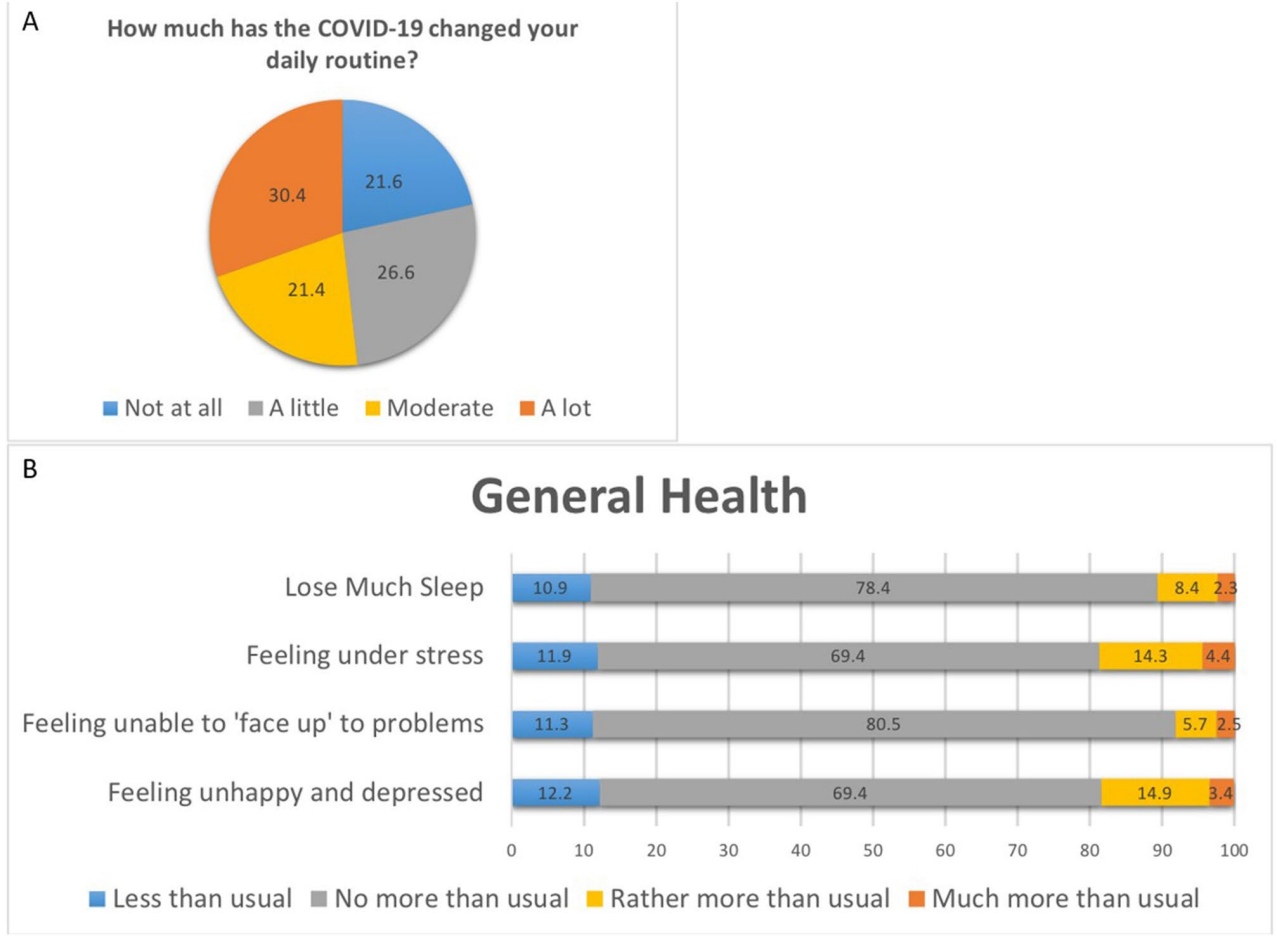
Impact of COVID-19 on respondents’ daily routine, general health, and well-being.

Although 30.4% of respondents (n = 159) reported that COVID-19 had caused their daily routine to “change a lot” (Fig. 2a), 69.4% (n = 363) of the participants did not experience more stress and unhappiness. Similarly, 78.4% (n = 410) reported not losing sleep more than usual and 80.5% (n = 421) participants responded that they were not experiencing more difficulties in facing up to problems encountered with COVID-19 (Fig. 2b; Table 2e). Multivariable analyses further showed in Table 3 that older participants were less likely to report change in daily routine (per 5 years older, OR = 0.75; P = 0.004), whilst participants with higher income were more likely (OR = 3.06; P = 0.012) to experience change in daily routine. Compared to Malays, Indians were less likely to experience change in daily routine (OR = 0.37, P = 0.002); conversely, Chinese were more likely (OR = 2.28, P = 0.028) to perceive so. On the other hand, compared to Malays, Indian (OR = 2.93, P = 0.003) and Chinese elderly (OR = 6.78, P < 0.001) were more likely to perceive that their well-beings were affected.

### Assessment on individual acceptance towards digital health

**Figure 3:**
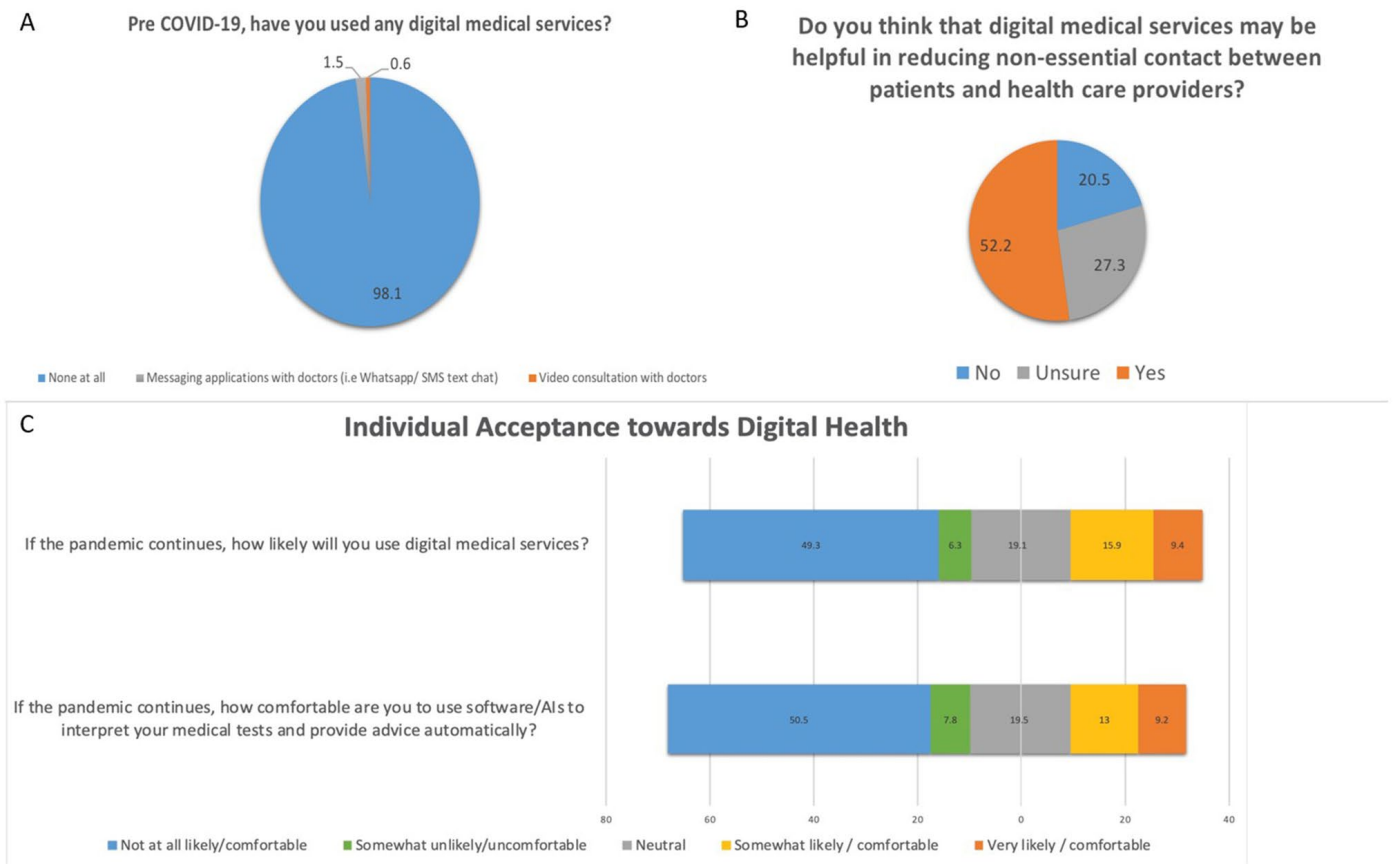
Respondents’ familiarity, attitude and acceptance towards the digital health services or technologies.

Of the participants, 98.1% (n = 513) reported not using any digital medical services for doctor consultation or medical follow-ups prior COVID-19 outbreak; and 47.8% (n = 250) responded “no” or “unsure” that digital health services would be helpful in reducing non-essential contact between patients and doctors (Fig. 3). A total of 55.6% participants (n = 291) reported either “not at all likely”, or “somewhat unlikely” in using these digital medical services if COVID-19 pandemic were to continue (Fig. 3c; Table 2f). Multivariable analysis shown in Table 5 presented that, compared to Malay participants, Indian participants were less likely to perceive that digital medical services were helpful in reducing non-essential contact (OR = 0.45; P = 0.003). Participants with higher income (OR = 1.96, P = 0.019) and higher educational level (OR = 2.58, P = 0.035) were more likely to perceive that such digital services were helpful. Furthermore, higher income individuals were also more receptive (OR = 2.58; P = 0.001) to use digital medical service if the pandemic were to continue. Finally, older participants were less receptive to use digital medical service (per 5 years older, OR = 0.71; P < 0.001).

**Table 5:** Association between demographic, socio-economic factors and medical history with Digital Health Acceptance.

Factors	Acceptance towards Digital Health			
	Agree that digital medical services are helpful in reducing non-essential contact		Likely to use digital medical services*	
	OR (95%CI)	P	OR (95%CI)	P
Age, per 5 years old	0.97 (0.83–1.13)	0.658	**0.71 (0.59–0.86)**	< 0.001
Female gender	0.81 (0.52–1.27)	0.362	0.96 (0.57–1.61)	0.874
**Ethnicity**				
Malay	Reference		Reference	
Indian	**0.45 (0.27–0.76)**	0.003	1.83 (0.98–3.43)	0.060
Chinese	1.30 (0.77–2.18)	0.321	1.59 (0.85–2.98)	0.149
**Income Category**				
< SGD $2000	Reference		Reference	
≥ SGD $2000	**1.96 (1.12–3.46)**	0.019	**2.58 (1.46–4.56)**	0.001
**Education**				
No Formal Education	Reference		Reference	
Formal Education^#^	**2.58 (1.07–6.22)**	0.035	2.24 (0.49–10.26)	0.298
**Housing Category**				
1–2 room public housing flat	Reference		Reference	
3–4 room public housing flat	1.98 (0.79–4.95)	0.143	1.77 (0.47–6.68)	0.403
≥ 5-room public housing flat	2.48 (0.92–6.64)	0.072	2.68 (0.68–10.62)	0.160
Current smoking status, yes	1.23 (0.59–2.56)	0.587	0.86 (0.37–1.99)	0.729
Living alone, yes	0.83 (0.35–1.98)	0.672	0.76 (0.25–2.38)	0.642
History of any chronic systemic diseases^+^, yes	1.35 (0.64–2.84)	0.435	1.73 (0.74–4.05)	0.209

SGD = Singapore Dollar.

*Answered either ‘Somewhat likely’ or ‘Very likely’ for the question on likelihood of using digital medical services.

^#^Defined as having primary or higher education.

^+^Defined as having diabetes, hypertension, chronic kidney disease, cardiovascular disease or hyperlipidaemia.

## Discussion

In this multi-ethnic Asian elderly group, we evaluated the pandemic’s impact on the participant’s well-being, as well as their knowledge and awareness of COVID-19. Overall, there was good consensus that COVID-19 is a serious public health threat with majority of participants showed high level of awareness on the asymptomatic transmission nature of COVID-19, as well as the relevant preventive measures to mitigate transmission. However, relatively few respondents were receptive to digital health services as an alternate means for medical consultation if the pandemic were to continue. This study presents present novel data on the perceptions of a multi-ethnic Asian population on COVID-19. These findings will be useful for policy makers to examine the effectiveness of current public communication strategies related to COVID-19. The observed findings also highlight the need to improve digital health acceptance and adoption among elderly as we move towards a new normal, post COVID-19.

Similar to our study, in a cross-sectional survey conducted by Wolf et al. in the US among adults with chronic condition during the onset of the pandemic^4^, most of the US participants were aware of the symptoms and prevention methods for COVID-19. A stark difference, however, is the response on preparedness for the outbreak. In the US study, around 20% of respondents stated they were prepared for the pandemic and around 10% of them indicated their confidence in the government’s ability to handle COVID-19. Similarly, in a study done in Russia, only 15% of the study population felt that their country was well-prepared and more than 50% of the population reported that they had low trust in the government and local authorities despite being well-informed on COVID-19 measures^23^. Our study showed that close to 70% of respondents were confident in the Singapore government’s ability to limit the widespread of COVID-19, with more than 60% of respondents indicated that they were prepared to handle a similar pandemic. Given that the US team found that the low rate of confidence was associated with a low health literacy and individuals who belonged to a non-White race, this exacerbates the fact that health literacy is a key to understanding government advisories as well as mediating the impact that the pandemic has on an individual’s mind set and health^1^.

Additionally, health literacy includes one’s ability to receive information and recognize whether they have been affected. A study done by Okan et al. on German participants aged 16 years and above with a mean age of 45.6 years, found that around 40% of respondents found it hard to identify information on how to recognize COVID-19 infections and majority of them felt confused by the information presented^15^. This adds to the perennial problem of poor readability of health information in various sources including the internet^24^ and impacts one’s health literacy. It also relates to the respondents’ perception on the reliability of their sources, indicating the importance of gaining the trust and confidence of the public in the course of the pandemic. With lower health literacy, patients lack the ability to understand the seriousness of the pandemic and the preventive measurements needed to take to prevent contracting the disease. In Singapore’s context, the high rate of COVID-19 awareness could be attributed to the effective dissemination of COVID-19 related information and knowledge in Singapore during the early phase of pandemic^25^. While most people are acting in a socially responsible way, there is still a small percentage of people ignoring preventive measurements and protective behaviour due to the lack of knowledge and awareness towards COVID-19^26^. It is therefore important that the public, especially the elderly, to have some degree of awareness and knowledge on COVID-19.

In our study, we found that COVID-19 caused an increase in respondents’ stress and reduced happiness level as compared to their sleeping habits and resilience level. In comparison to studies conducted by Kivi et al. on the Swedish population and van Tilburg et al. on the Netherlands population, respondents indicated an equally high level of wellbeing as compared to previous years^27,28^. It is important to note that the Netherlands citizens had to practice social distancing but not social isolation, and the Swedish older adults were still carrying on with their daily activities as the study was conducted during the initial part of the pandemic. Our study was conducted during the lockdown period in Singapore, where citizens had to stay at home for a prolonged period, which could be an explanation for the difference in wellbeing results. The comparison of our results to the mentioned studies is a good indicator that wellbeing should be monitored closely among the elderly especially since there are other factors such as government policies and lifestyle factors that could play a role.

In terms of ethnicity differences, compared to Malays, Indian participants were five times less worried about getting COVID-19 (Table 3), and about two times were less aware that a COVID-19 infected person can be asymptomatic (Table 4). Compared to Malays and Chinese, there were more Indian individuals who reported that they had no changes in their daily routines compared to Malay (Indian 33.3% and Malay 22.0% respectively) and Chinese (9.7% respectively) participants (Supplementary Table 1e). In contrast, about six times more Chinese participants felt that their well-being had been affected by COVID-19 and three times more Chinese felt less prepared compared to Malay and Indian participants (Table 3). These results suggest that less knowledge, awareness and concern of COVID-19 and the low COVID-19 death rate in Singapore could lead to optimism bias which makes individuals less likely to change their actions or behaviour to curb the spread of disease^29,30^. It is important for government bodies to understand and evaluate if there is a knowledge gap on the current health issues among different ethnicities and rectify the problem promptly.

The first outbreak that Singapore had to manage in the twenty-first century was the SARS pandemic in 2003^31^. Post-SARS crisis, Deurenberg-Yap et al., found that Singaporean adults had low knowledge score on SARS and control measures even though they expressed a high level of public trust in the government^31^. Fast forward to 2020 during the COVID-19 pandemic, we found high government trust and good awareness and compliance with public health measures. The relative increase in public knowledge levels over the years could be a testament to the government efforts in being more deliberate with raising awareness and the public’s efforts in being more informed about COVID-19.

The COVID-19 pandemic had caused a massive acceleration in the adoption of digital technology with many clinical facilities reduced or even ceased. Healthcare providers had also remodelled physical services to encourage patients to seek care online instead^32,33^. The use of digital technology had been described as “electronic personal protective equipment” (e-PPE) and had brought upon advantages including convenience, less exposure from physical interactions and reduced cost^34^. However, it is important to consider patients’ acceptance when addressing the feasibility and sustainability of digital solutions in healthcare. Our findings showed that almost all participants did not use digital health services prior to COVID-19 pandemic, which could be explained by the lack of digital literacy and understanding of these services. This corroborates with an earlier study that reported the elderly had reduced patient acceptance of mobile technology (MT) in emergency services^35^. Almost half of elderly respondents perceive that these services are not helpful in reducing non-essential contact in clinic setting and have less acceptance to such services. In contrast, our findings indicated that participants with higher income and formal education do find digital health services useful and were more likely to use the services compared to participants of lower income and without formal education. It showed that individuals of lower income, less resources and lower education level have lower acceptance of digital solutions in healthcare^36^. This highlights the crucial need for administrators and providers to address health care accessibility and public health awareness among underprivileged individuals as the pandemic situation persists.

Our study had several strengths. First, our study sample was of substantial size, and comprised of the three main ethnic groups in Asia. Secondly, our data were less prone to recall bias and response error as the survey was conducted while the roll-out of COVID-19 measures were still fairly recent back then. Thirdly, in our multivariable analyses, we were able to control for multiple relevant confounders including demographic, social economic status and systemic comorbidities. However, our study has a few limitations. First, this questionnaire study was conducted on a selected group of SEED study, thus potentially introducing selection bias. In addition, while we assessed digital acceptance in the questionnaire, we did not further evaluate participants’ digital literacy level, a factor which might influence one’s acceptance of digital health services. Lastly, our findings only captured responses during the initial stage of the pandemic. The responses and behaviour captured then, may continue to change with time as the pandemic situation evolves.

In conclusion, in this multi-ethnic Asian study, we observe generally good awareness and knowledge of COVID-19 in elderly. However, the acceptance towards digital health services among elderly remains low, amidst the pandemic. Therefore, more work will be needed to improve digital health acceptance and adoption as we navigate the new normal post COVID-19.

## Supplementary Information

Supplementary Information.
