# Supplementary material for: COVID-19 awareness, knowledge and perception towards digital health in an urban multi-ethnic Asian population
Source: Sci Rep. 2021 May 24;11:10795. doi: 10.1038/s41598-021-90098-6 (PMC8144201; doi:10.1038/s41598-021-90098-6)

COVID-19 Awareness, Knowledge and Perception towards Digital Health in an Urban Multi-Ethnic Asian Population.

Cong Ling Teo, BSc[1*], Miao Li Chee, BSc[1*], Kai Hui Koh, BSc[1], Rachel Marjorie Wei Wen Tseng, BA[1], Shivani Majithia, OD[1], Sahil Thakur, MBBS[1], Dinesh Visva Gunasekeran, MD[3], Simon Nusinovici, PhD[1], Charumathi Sabanayagam MD PhD[1,2], Tien Yin Wong, MD PhD[1,2,3], Yih-Chung Tham, PhD[1,2*] Ching-Yu Cheng, MD MPH PhD [1,2,3*]

[1] Singapore Eye Research Institute, Singapore National Eye Centre, Singapore.

[2] Duke-NUS Medical School, Singapore.

[3] Department of Ophthalmology, Yong Loo Lin School of Medicine, National University of Singapore and National University Health System, Singapore.

*These authors contributed equally: Cong Ling Teo & Miao Li Chee. These authors jointly supervised this work: Yih-Chung Tham & Ching-Yu Cheng.

Running title: Health Literacy and Digital acceptance amidst COVID-19 among Elderly Asians.

Keywords: COVID-19; Health Literacy; Digital Acceptance; Asians.

Correspondence author:

Dr Yih-Chung Tham & Professor Ching-Yu Cheng
Singapore Eye Research Institute, The Academia, 20 College Road, Discovery Tower Level 6, Singapore, 169856, Singapore.

E-mail: tham.yih.chung@seri.com.sg & chingyu.cheng@duke-nus.edu.sg.
Tel: +65 6576 7277; **Fax:** +65 6576 7277

Supplementary Table 1: Knowledge, attitude, preparedness and digital health acceptance toward COVID-19 in each ethnic group.

	Malays (n=177)	Indians (n=171)	Chinese (n=175)	P
**(a) COVID-19 awareness and concern**				
**On a scale of 1 to 10, how serious of a public health threat do you think the COVID-19 is or might become? (1 being no threat at all, 10 being a very serious public health threat)**	8 ± 1.8	7 ± 3	7.6 ± 2.3	<0.001
**How worried are you about getting the COVID-19?**				
Very worried	63 (35.6)	49 (28.7)	63 (36.0)	<0.001
A little worried	82 (46.3)	25 (14.6)	77 (44.0)	
Not worried at all	32 (18.1)	97 (56.7)	35 (20.0)	
**Do you think that you will get sick from the COVID-19?**				
I definitely will	24 (13.6)	18 (10.5)	39 (22.3)	<0.001
I probably will	38 (21.5)	26 (15.2)	49 (28.0)	
Neutral	75 (42.4)	16 (9.4)	47 (26.9)	
Not at all	40 (22.6)	111 (64.9)	40 (22.9)	
**How likely do you think it is that someone you know may get sick from the COVID-19 this year?**				
Very likely	11 (6.2)	10 (5.8)	13 (7.4)	<0.001
Somewhat likely	20 (11.3)	17 (9.9)	23 (13.1)	
Neutral	86 (48.6)	6 (3.5)	64 (36.6)	
Somewhat unlikely	24 (13.6)	4 (2.3)	32 (18.3)	
Not at all likely	36 (20.3)	134 (78.4)	43 (24.6)	
**(b) COVID-19 knowledge**				
**Are you aware that COVID-19 carriers can be asymptomatic? For example, absence of running nose, cough, fever or appearing to be fine.**				

Yes	139 (78.5)	114 (66.7)	142 (81.1)	0.013
Unsure	23 (13.0)	37 (21.6)	17 (9.7)	
No	15 (8.5)	20 (11.7)	16 (9.1)	
**On a scale of 1 to 10, how likely do you think a person who is infected by COVID-19 will display no symptoms or mild symptoms? e.g. mild cough, itchy throat and mild fever. (1 being not at all likely, 10 being a very likely)**	5.3 ± 1.8	4.8 ± 2.5	5.8 ± 2.4	<0.001
**On a scale of 1 to 10, how likely do you think a person who get COVID-19 will die as a result? (1 being not at all likely, 10 being a very likely)**	6.3 ± 2	3.4 ± 2.8	5.5 ± 2.5	<0.001
**Which of the following do you think are important prevention methods for the COVID-19?:**				
**Wear a mask (as long as you leave the house)**				
Yes	174 (98.3)	168 (98.2)	169 (96.6)	0.199
Unsure	0 (0.0)	0 (0.0)	3 (1.7)	
No	3 (1.7)	3 (1.8)	3 (1.7)	
**Keep a minimum of 1m apart from other public members**				
Yes	171 (96.6)	166 (97.1)	165 (94.3)	0.224
Unsure	0 (0.0)	1 (0.6)	4 (2.3)	
No	6 (3.4)	4 (2.3)	6 (3.4)	
**Wash your hands frequently**				
Yes	175 (98.9)	167 (97.7)	167 (95.4)	0.233
Unsure	1 (0.6)	0 (0.0)	2 (1.1)	
No	1 (0.6)	4 (2.3)	6 (3.4)	
**Stay at home as much as possible**				
Yes	168 (94.9)	165 (96.5)	159 (90.9)	0.105

Unsure	3 (1.7)	0 (0.0)	6 (3.4)	
No	6 (3.4)	6 (3.5)	10 (5.7)	
**Avoid touching your face, eyes, nose and mouth**				
Yes	171 (96.6)	153 (89.5)	159 (90.9)	0.004
Unsure	3 (1.7)	2 (1.2)	8 (4.6)	
No	3 (1.7)	16 (9.4)	8 (4.6)	
**(c) Information sources on COVID-19**				
**Where do you get information about COVID-19? (Check all that apply)**				
TV	162 (91.5)	149 (87.1)	161 (92.0)	0.244
Newspaper	82 (46.3)	60 (35.1)	118 (67.4)	<0.001
Radio	85 (48.0)	50 (29.2)	42 (24.0)	<0.001
Family and Friends	100 (56.5)	29 (17.0)	79 (45.1)	<0.001
Posters/Leaflets	3 (1.7)	2 (1.2)	24 (13.7)	<0.001
Hospital/Polyclinic/GP	1 (0.6)	0 (0.0)	13 (7.4)	<0.001
Internet/ Sharing on Social Media (Facebook/Twitter/etc)	35 (19.8)	32 (18.7)	69 (39.4)	<0.001
Sharing on messaging applications (Whatsapp/ etc)	18 (10.2)	32 (18.7)	70 (40.0)	<0.001
**(d) Preparedness**				
**How confident are you that Singapore government can prevent a further widespread outbreak in our local community?**				
Very confident	123 (69.5)	136 (79.5)	104 (59.4)	<0.001
Somewhat confident	36 (20.3)	12 (7.0)	43 (24.6)	
Neutral	17 (9.6)	15 (8.8)	24 (13.7)	
Not very confident	0 (0.0)	5 (2.9)	3 (1.7)	
Not confident at all	1 (0.6)	3 (1.8)	1 (0.6)	
**How prepared do you think you are if there were to be a further widespread outbreak in the local community?**				

Very prepared	27 (15.3)	126 (73.7)	40 (22.9)	<0.001
Somewhat prepared	73 (41.2)	19 (11.1)	61 (34.9)	
Neutral	69 (39.0)	16 (9.4)	53 (30.3)	
Not very well prepared	3 (1.7)	8 (4.7)	11 (6.3)	
Not prepared at all	5 (2.8)	2 (1.2)	10 (5.7)	
**(e) General Health, well-being Questionnaire**				
**How much has the COVID-19 changed your daily routine?**				
A lot	39 (22.0)	55 (32.2)	65 (37.1)	<0.001
Moderate	28 (15.8)	31 (18.1)	53 (30.3)	
A little	71 (40.1)	28 (16.4)	40 (22.9)	
Not at all	39 (22.0)	57 (33.3)	17 (9.7)	
**Lose much sleep**				
Less than usual	50 (28.2)	3 (1.8)	4 (2.3)	<0.001
No more than usual	123 (69.5)	153 (89.5)	134 (76.6)	
Rather more than usual	4 (2.3)	12 (7.0)	28 (16.0)	
Much more than usual	0 (0.0)	3 (1.8)	9 (5.1)	
**Feel under stress**				
Less than usual	53 (29.9)	6 (3.5)	3 (1.7)	<0.001
No more than usual	110 (62.1)	138 (80.7)	115 (65.7)	
Rather more than usual	8 (4.5)	18 (10.5)	49 (28.0)	
Much more than usual	6 (3.4)	9 (5.3)	8 (4.6)	
**Feel unable to 'face up' to problems encountered**				
Less than usual	53 (29.9)	4 (2.3)	2 (1.1)	<0.001
No more than usual	119 (67.2)	160 (93.6)	142 (81.1)	

Rather more than usual	4 (2.3)	4 (2.3)	22 (12.6)	
Much more than usual	1 (0.6)	3 (1.8)	9 (5.1)	
**Feel unhappy and depressed**				
Less than usual	51 (28.8)	9 (5.3)	4 (2.3)	<0.001
No more than usual	113 (63.8)	138 (80.7)	112 (64.0)	
Rather more than usual	12 (6.8)	17 (9.9)	49 (28.0)	
Much more than usual	1 (0.6)	7 (4.1)	10 (5.7)	
**(f) Assessment on Individual Acceptance towards Digital Health**				
**Before the COVID-19 outbreak, have you used any of these 'digital medical services' for a doctor consultation or follow-up for test results? (Check all that apply)**				
None at all	174 (98.3)	169 (98.8)	170 (97.1)	0.501
Messaging applications with doctors (for example, Whatsapp/ SMS text chat)	2 (1.1)	2 (1.2)	4 (2.3)	0.607
Video consultation with doctors	1 (0.6)	0 (0.0)	2 (1.1)	0.371
**Do you agree that the digital medical services mentioned in the previous question may be helpful to reduce non-essential contact between patients and doctors/health care providers?**				
Yes	97 (54.8)	63 (36.8)	113 (64.6)	<0.001
Unsure	60 (33.9)	38 (22.2)	45 (25.7)	
No	20 (11.3)	70 (40.9)	17 (9.7)	
**In your opinion, if the COVID-19 pandemic continues, how likely will you use these digital medical services (video consultation with doctors, Whatsapp/ SMS text chat)**				
Very likely	9 (5.1)	23 (13.5)	17 (9.7)	<0.001
Somewhat likely	22 (12.4)	28 (16.4)	33 (18.9)	
Neutral	31 (17.5)	13 (7.6)	56 (32)	
Somewhat unlikely	11 (6.2)	13 (7.6)	9 (5.1)	

Not at all likely	104 (58.8)	94 (55.0)	60 (34.3)	
**If the COVID-19 pandemic continues, how comfortable are you to use software/AI systems to interpret your medical tests/scans and provide advice automatically?**				
Very comfortable	4 (2.3)	26 (15.2)	18 (10.3)	<0.001
Somewhat comfortable	13 (7.3)	27 (15.8)	28 (16.0)	
Neutral	40 (22.6)	15 (8.8)	47 (26.8)	
Somewhat uncomfortable	17 (9.6)	7 (4.1)	17 (9.7)	
Not at all comfortable	103 (58.2)	96 (56.1)	65 (37.1)	

Supplementary Figure 1: Summary of respondents' COVID-19 awareness and concern.

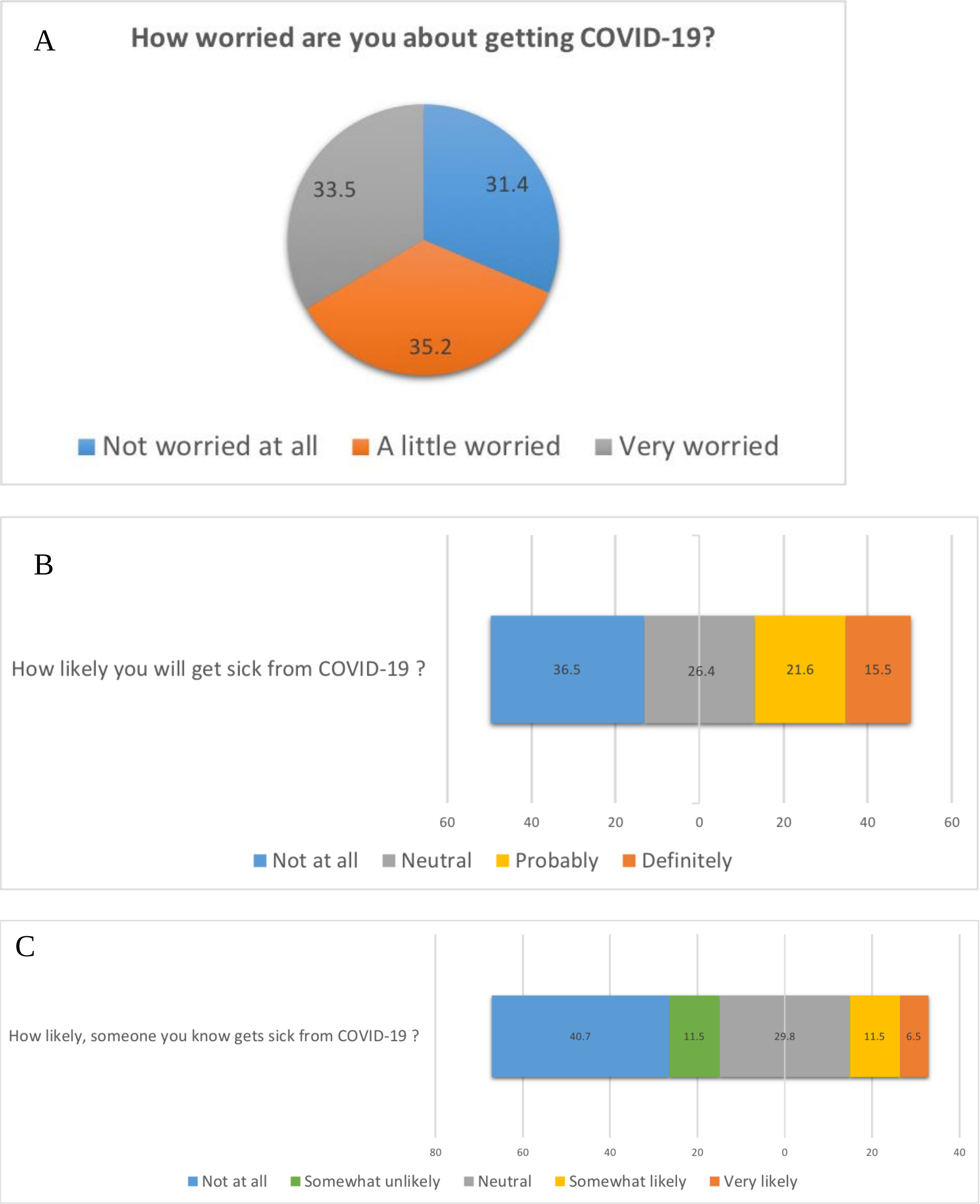

Supplementary Figure 2: Summary of Respondents' knowledge on COVID-19 symptoms and relevant safe practices.

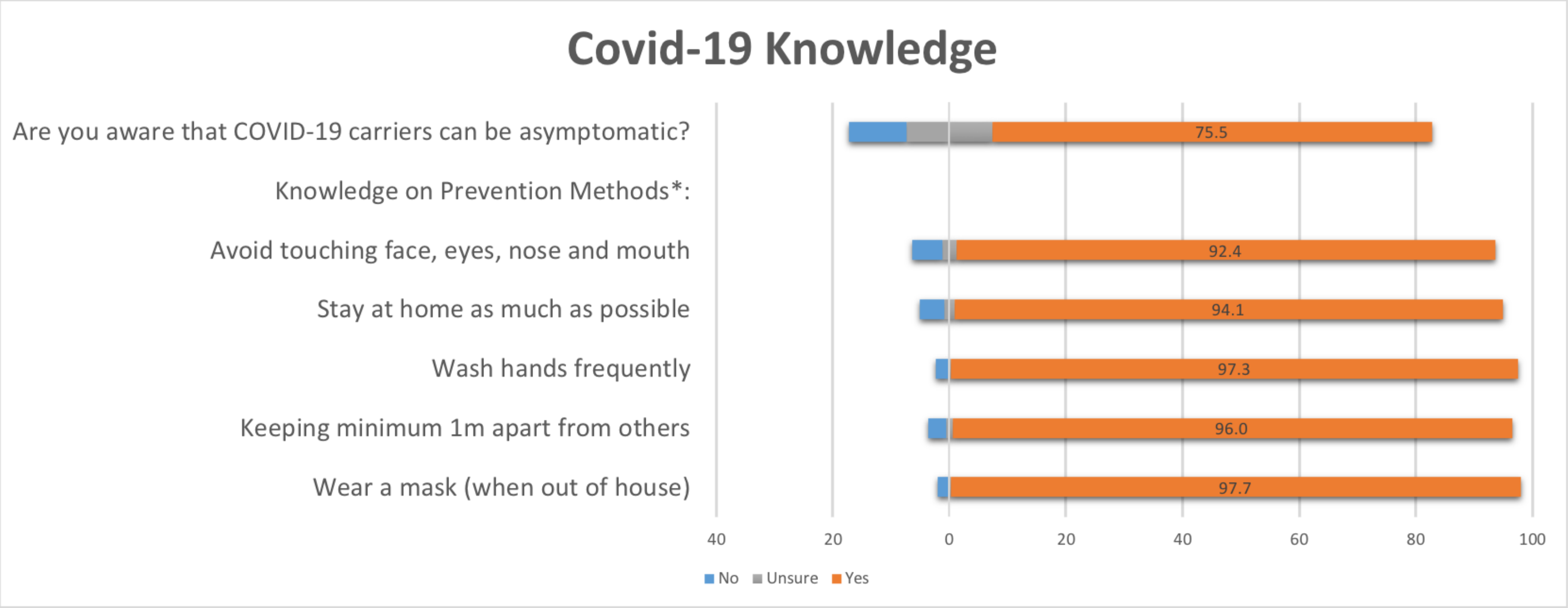

Supplementary Figure 3: Respondents' opinion on confidence in government preparedness and individual preparedness in the event of a future outbreak

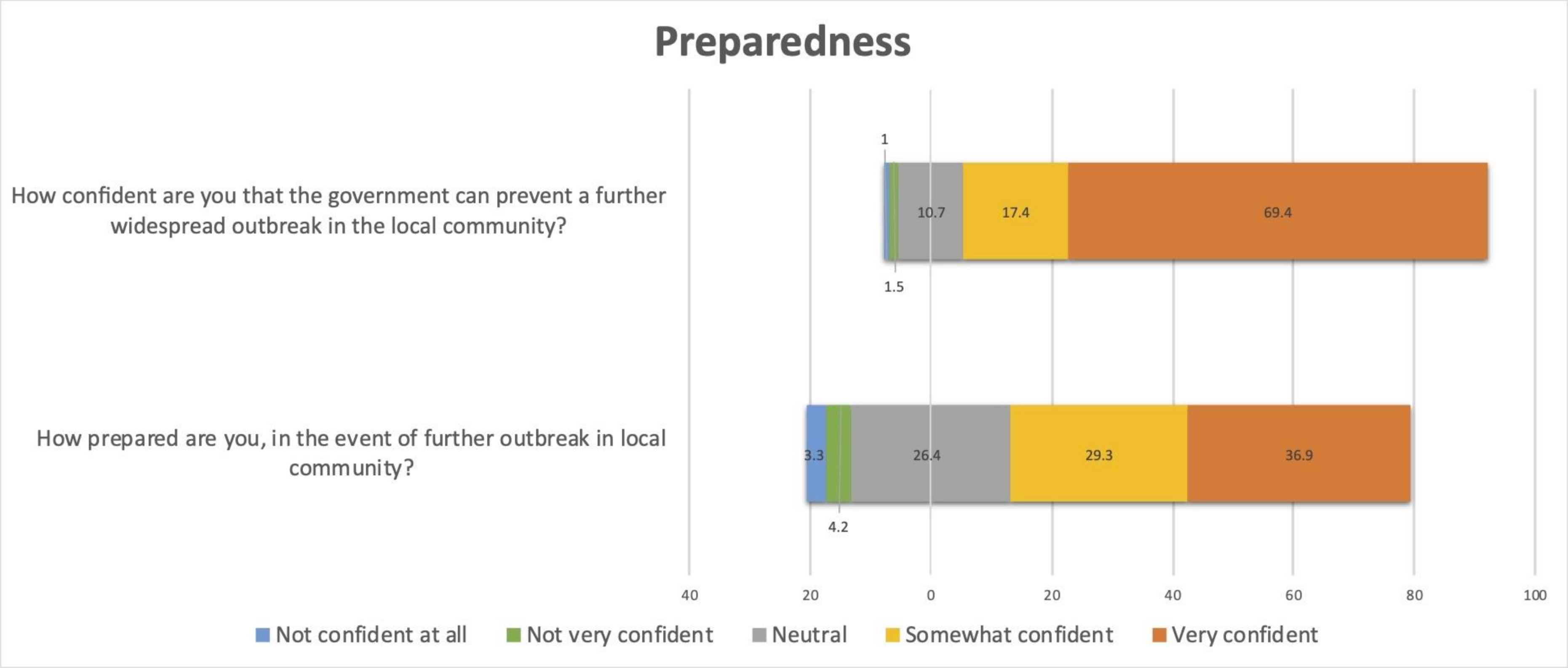

Supplement: Supplementary file 1 — Supplementary Information. [file 41598_2021_90098_MOESM1_ESM.pdf]
